# Efficacy and safety of rituximab in multiple sclerosis and neuromyelitis optica spectrum disorder

**DOI:** 10.1038/s41598-024-53838-y

**Published:** 2024-02-12

**Authors:** Tatchaporn Ongphichetmetha, Jiraporn Jitprapaikulsan, Sasitorn Siritho, Natthapon Rattanathamsakul, Thammachet Detweeratham, Naraporn Prayoonwiwat

**Affiliations:** 1 Division of Neurology, Department of Medicine, Faculty of Medicine Siriraj Hospital, Mahidol University, 2 Wanglang Rd, Siriraj, Bangkok noi, Bangkok, 10700 Thailand; 2 Siriraj Neuroimmunology Center, Faculty of Medicine Siriraj Hospital, Mahidol University, Bangkok, 10700 Thailand; 3 Division of Clinical Epidemiology, Department of Research and Development, Faculty of Medicine Siriraj Hospital, Mahidol University, Bangkok, 10700 Thailand; 4 Bumrungrad International Hospital, Bangkok, 10110 Thailand

**Keywords:** Neurology, Demyelinating diseases

## Abstract

In Thailand, resource limitations lead many multiple sclerosis (MS) and neuromyelitis optica spectrum disorder (NMOSD) patients to use off-label immunosuppressants. This study assesses the efficacy and safety of rituximab (RTX) with a CD19-based reinfusion regimen among Thai MS and NMOSD patients. A retrospective review of patients at the Faculty of Medicine Siriraj Hospital from January 1994 to April 2023 was conducted. The primary outcome assessed was the change in annualized relapse rate (ARR) for patients using RTX for over a year. Secondary outcomes included changes in the Expanded Disability Status Scale (EDSS) scores, time to the first relapse after RTX initiation for patients using RTX for over a year, and an evaluation of the safety of RTX. The study encompassed 36 MS and 39 NMOSD patients. A majority of patients (91.7% of MS and 79.5% of NMOSD) experienced no relapses during a median follow-up of 30 months (Interquartile range [IQR] 20–46) and 31 months (IQR 23–41), respectively. The median ARR significantly decreased in both MS (from 0.77 [IQR 0.42–1.83] to 0 [IQR 0–0], *p *< 0.001) and NMOSD (from 0.92 [IQR 0.68–1.78] to 0 [IQR 0–0.17], *p *< 0.001) patients after switching to RTX, with no difference between those following a fixed 6-month time point regimen and a CD19-based reinfusion regimen. Median EDSS scores improved significantly at the last follow-up visit in both groups. The mean time to the first subsequent relapse was 8.3 ± 3.0 months in MS and 6.8 ± 1.7 months in NMOSD. Mild adverse drug reactions occurred in 44% of patients. RTX effectively prevents relapses in Thai MS and NMOSD patients, with no observed serious adverse drug reactions.

## Introduction

Multiple sclerosis (MS) and neuromyelitis optica spectrum disorder (NMOSD) are two of the most prevalent central nervous system idiopathic inflammatory demyelinating diseases (CNS-IIDDs) in Thailand, which mainly affect the working-age population causing multiple domains of neurological deficits^1^. Given the socioeconomic status of the Thai people and the limited resources of the health system, most MS and NMOSD patients in Thailand cannot afford approved disease-modifying therapies (DMTs) from the Food and Drug Administration (FDA) and monoclonal antibodies, respectively^2^. Instead, Thai MS and NMOSD patients receive off-label medications, such as azathioprine and mycophenolate mofetil^2^.

Rituximab (RTX) is a chimeric monoclonal anti-CD20 antibody first developed for the treatment of B-cell lymphomas. Later, RTX is widely used in rheumatologic diseases^3,4^. The efficacy of RTX in NMOSD in the prevention of relapses has been reported in the phase II randomized controlled trial (RIN-1 study) and subsequent real-world observational studies^5–9^. Focusing on MS, growing evidence support that B lymphocytes have an important role in the pathophysiology of the disease^10–13^. Clinical trials and observational studies have consistently demonstrated the efficacy of RTX in preventing clinical relapses and reducing the occurrence of new gadolinium-enhancing or T2-weighted lesions in individuals with relapsing–remitting multiple sclerosis (RRMS)^14–22^. Therefore, RTX is used off-label nowadays in both MS and NMOSD around the world due to its cost-effectiveness and accessibility^16^. The dose and dosing interval vary between different centers^23–29^. In Thailand, a study reported the cost-effectiveness of RTX for MS and NMOSD, focusing on the fixed 6-month time point regimen^30,31^.

This study aimed to report on the real-world efficacy and safety of RTX in MS and NMOSD in Thailand, a resource-limited country. We also emphasize the robust efficacy of RTX in the extended dosing interval.

## Methods

### Study design and participants

This study was a retrospective cohort study conducted in two hospitals in Thailand, Siriraj Hospital, a tertiary university hospital, and Siriraj Piyamaharajkarun Hospital, a large-scale private hospital. Patients with MS or NMOSD with aquaporin-4 immunoglobulin G (AQP4-IgG) treated with RTX from January 1994 to January 2023 were identified through the Siriraj CNS-IIDDs registry. The inclusion criteria were (1) patients diagnosed with MS according to the 2017 McDonald criteria^32^ or NMOSD with AQP4-IgG according to the 2015 international consensus diagnostic criteria for NMOSD^33^ and (2) had a history of RTX therapy for more than a year. Patients who were concurrently treated with other DMTs or immunosuppressants (IS) except corticosteroids, were excluded. The study protocol was approved by the Siriraj Institutional Review Board (COA no. Si 057/2023) and all methods were performed in accordance with the guidelines and regulations. Informed consent was obtained from all participants for the use of de-identified medical records.

### Demographic and clinical data

#### Before RTX initiation

Data, including sex, age at the time of disease onset and diagnosis, diagnosis, clinical symptoms at onset, total number of relapses before diagnosis, previous DMTs or IS, total number of relapses during previous treatments, total number of relapses one year before RTX initiation, baseline Expanded Disability Status Scale (EDSS) scores before RTX initiation (assessed at remission or at least three months after relapses), reasons for treatment switching to RTX, and indications for RTX, were collected. Magnetic resonance imaging (MRI) of the brain and spine within 12 months prior to RTX initiation was reviewed for the presence of gadolinium enhancing lesions (Gd+ lesions) in MS patients.

#### After RTX initiation

The total number with detailed relapses during RTX treatment and EDSS scores at the last follow-up visit were recorded. Any adverse drug reactions, as defined by the World Health Organization^34^, were documented. The majority of MS patients receiving RTX underwent brain and spinal MRI assessments at least two times, including a re-baseline MRI at 6 months and another at more than 12 months after the initiation of RTX treatment to assess MRI activity. All available brain and spinal MRI during RTX treatment in MS patients were reviewed with board-certified neuroradiologists for the presence of new Gd+ lesions, new T2-weighted hyperintense lesions, and enlarged T2-weighted hyperintense lesions, with a comparison between the re-baseline MRI and subsequent MRI.

#### Induction and maintenance treatment regimens

In this study, both the original formulation of RTX and its biosimilar were prescribed. The induction regimen of RTX for MS and NMOSD patients mostly consisted of two infusions of 1000 mg each, administered 15 days apart. For maintenance treatment regimens, the fixed 6-month time point regimen (characterized by scheduling RTX infusions every 6 months, irrespective of CD19+ B lymphocyte levels) or a CD19-based reinfusion regimen (distinguished by scheduling RTX infusions based on CD19+ B lymphocyte level assessment before each maintenance cycle), was applied. The CD19-based reinfusion regimen was defined as reinfusion when CD19+ B lymphocyte level was more than 1% of mononuclear cells. Acetaminophen 500 mg, with diphenhydramine 50 mg, were administered orally 30 min before infusion of RTX as pretreatment medications. Intravenous dexamethasone and chlorpheniramine were optional.

#### CD19+ *B lymphocyte subpopulation measurement*

Measurement of the CD19+ B lymphocyte subpopulation, including both absolute CD19+ B lymphocyte counts and percentages, is routinely performed in clinical practice in all patients receiving RTX before their first RTX cycle and is monitored every 3 to 6 months, depending on the treating physicians. If the dosing interval is extended to more than 6 months, CD19+ B lymphocyte level is normally checked every 1 month thereafter. The subpopulation of CD19+ B lymphocytes was measured using flow cytometry.

### Outcome measurements

Relapse is defined as newly developing neurological symptoms or reactivation of preexisting neurological deficits with objective findings reflecting a central nervous system demyelinating event persisting for a minimum of 24 h in the absence of fever or infection^19^. The annualized relapse rate (ARR) was calculated as total number of relapses per person-years during the observation period^9^. No evidence of disease activity (NEDA-3) in MS was a composite score comprising absence of clinical relapses, disability progression, and new MRI disease activity (new Gd+ lesions, new T2-weighted hyperintense lesions, or enlarged T2-weighted hyperintense lesions)^35^.

The efficacy outcomes focused on preventing clinical relapses in MS and NMOSD patients, reducing MRI activity, and stabilizing disability progression in MS patients. Therefore, the primary outcomes included the difference in ARR before and after RTX treatment. It is noteworthy that ARR was exclusively calculated for individuals with a disease duration exceeding one year before RTX initiation to mitigate the risk of overestimating ARR. Secondary outcomes encompassed the time to the first relapse, the proportion of relapse-free patients, the proportion of patients achieving NEDA-3, and the difference in EDSS scores before and after RTX treatment. The assessment of the proportion of patients experiencing any adverse drug reactions after RTX initiation was considered a secondary outcome.

### Statistical analysis

Continuous variables were reported as the mean with standard deviation (SD) for normally distributed data and the median with interquartile range (IQR) for nonnormally distributed data. Categorical variables were reported as a percent proportion. For inferential statistics, the Wilcoxon signed rank test was used to compare outcomes between pre and post RTX conditions. The statistical significance level was set at a *p* value of less than 0.05. Kaplan–Meier curves were also used to visualize relapse-free survival rates using the time from the first cycle of RTX infusion to the first post RTX relapse.

Statistical analyzes and data processing were performed using PASW Statistics for Windows version 18.0 (SPSS Inc., Chicago, IL, USA) and PRISM version 9.0 (GraphPad, SanDiego, CA, USA).

### Subgroup analyzes

Subgroup analyzes were planned and conducted using two factors: 1) maintenance treatment regimens (a fixed 6-month time point regimen subgroup or a CD19-based reinfusion regimen subgroup, and 2) history of previous DMTs or IS (a treatment-naïve subgroup or a treatment-experienced subgroup). Analyses were performed separately for MS and NMOSD patients. The differences between the pre and post RTX ARR in each maintenance treatment regimen subgroup, and the differences between the pre and post RTX number of relapses in a treatment-naïve and a treatment-experienced subgroup were evaluated and compared using the Mann–Whitney U test to determine the robustness of the efficacy outcomes.

We further examined the average dosing interval of RTX by calculating the mean of dosing intervals after the induction regimen for each patient. This analysis aimed to assess the efficacy of RTX when extending the dosing interval beyond 6 months during the maintenance phase.

## Results

Seventy-five MS and NMOSD patients who received RTX for more than a year were included (Table 1). Of these, 36 (48%) were MS and 39 (52%) were NMOSD with AQP4-IgG. Among 36 MS patients, 33 (91.7%) had relapsing–remitting MS and three had secondary progressive MS. The mean (± SD) ages at the time of onset were 27.9 ± 12.3 years in MS and 42.0 ± 14.0 years in NMOSD. Twenty-five (69.4%) MS and thirty-three (84.6%) NMOSD patients were female. The median of EDSS scores before receiving RTX was 4.5 (IQR 3.0–6.0) in NMOSD patients and 2.0 (IQR 0.0–4.0) in MS patients. Concentrating on disease activity 1 year before RTX initiation, the median number of relapses was 1 (IQR 0.3–1) and 1 (IQR 1–2) in MS and NMOSD, respectively. Thirteen of 31 MS patients (41.9%) with available pre RTX MRI data had gadolinium enhancing lesions.

**Table 1:** Baseline characteristics of Thai MS and NMOSD patients receiving rituximab (RTX).

Characteristics	MS patients (n = 36)	NMOSD patients (n = 39)
Age at onset, years, mean ± S.D	27.9 ± 12.3	42.0 ± 14.0
Female sex, n (%)	25 (69.4)	33 (84.6)
Disease duration before RTX, years, median (IQR)	4.9 (1.8–8.0)	2.9 (0.7–6.9)
Total number of relapses before RTX, median (IQR)	3 (2–5)	3 (2–6)
Number of relapses 1 year before RTX; median (IQR)	1 (0.3–1)	1 (1–2)
EDSS scores before RTX, median (IQR)	2.0 (0.0–4.0)	4.5 (3.0–6.0)
Treatment-naïve, n (%)	11 (30.6)	13 (33.3)
Treatment-experienced, n (%)	25 (69.4)	26 (66.7)
DMTs or IS ever received prior to RTX, n (%)		
Azathioprine	16 (64.0)	23 (88.5)
Mycophenolate mofetil	8 (32.0)	14 (53.8)
Methotrexate	1 (4.0)	0 (0.0)
Mitoxantrone	0 (0.0)	1 (3.8)
Cyclophosphamide	0 (0.0)	1 (3.8)
Interferon-β	3 (12.0)	1 (3.8)
Teriflunomide	3 (12.0)	0 (0.0)
Fingolimod	2 (8.0)	0 (0.0)
Reasons for RTX initiation, n (%)		
Ineffectiveness of DMTs or IS	16 (44.4)	15 (38.5)
Adverse drug reactions from DMTs or IS*	2 (5.6)	5 (12.8)
High disease activity^†^	7 (19.4)	0 (0.0)
Financial issue	7 (19.4)	6 (15.4)
Patient’s preference	4 (11.1)	13 (33.3)
Induction regimen; n (%)		
Two 1000 mg infusion 15 days apart	33 (91.7)	31 (79.5)
Two 500 mg infusion 15 days apart	0 (0)	1 (2.6)
500 mg on day 1 and 1000 mg on day 15	0 (0)	2 (5.1)
1000 mg on day 1 and 500 mg on day 15	0 (0)	1 (2.6)
Single 1000 mg infusion	3 (8.3)	4 (10.3)
Maintenance regimen; n (%)		
A fixed time point (6 months) regimen	7 (19.4)	15 (38.5)
A CD19-based reinfusion regimen	29 (80.6)	24 (61.5)
Duration of RTX^‡^, months, median (IQR)	30 (20–46)	31 (23–41)
Total cycles of RTX; median (IQR)	6 (4–7.8)	7 (5–8)
Average dosing interval of RTX, months; median (IQR)	6.8 (6.3–7.5)	6.4 (6.0–7.8)

*DMT* Disease-modifying therapy, *EDSS* Expanded Disability Status Scale, *IS* Immunosuppressants, *MRI* Magnetic resonance imaging, *MS* Multiple sclerosis, *NMOSD* Neuromyelitis optica spectrum disorder, *RTX* Rituximab.

*Adverse drug reactions included leukopenia (white blood cell count < 3000/μL), neutropenia (absolute neutrophil count < 1500/μL), lymphopenia (absolute lymphocyte count < 1000/μL), transaminitis, gastrointestinal disturbance, infusion reactions, infection, alopecia, cardiac arrhythmia, etc.

^†^Some patients with high disease activity received off-label rituximab as the first agent due to its cost-effectiveness.

^‡^The median duration of RTX was equal to the median follow-up period, because all patients continued RTX until the end of the study period.

Eleven MS (30.6%) and 13 NMOSD (33.3%) patients received RTX as the first-line therapy (treatment-naïve). The rest had received prior DMTs/IS. The previous DMTs/IS ever received were listed in Table 1. The median of the last dose of azathioprine was 100 mg/day (range 75–200) among MS patients and 112.5 mg/day (range 50–200) among NMOSD patients, while MMF was discontinued at the last dose of 1500 mg/day (range 500–2500) in MS patients and 1500 mg/day (range 250–3000) in NMOSD patients.

### Rituximab treatment summary

RTX treatment is summarized in Table 1. Approximately 90% of MS patients and 80% of NMOSD patients received the induction regimen with two 1000 mg infusions 15 days apart. The selection of maintenance treatment regimens depended on the treating physicians and CD19+ B lymphocyte level after the first cycle. Twenty-nine (80.6%) of MS patients and 24 (61.5%) of NMOSD patients were scheduled for RTX with adjusted intervals by CD19+ B lymphocyte level (a CD19-based reinfusion regimen). The remainder followed a fixed 6-month time point regimen. The median average dosing interval was 6.8 months (IQR 6.3–7.5) in MS patients and 6.4 months (IQR 6.0–7.8) in NMOSD patients. Fifty-eight patients, comprising 32 MS patients and 26 NMOSD patients, had an extended average dosing interval of rituximab for more than 6 months. There were 5 NMOSD patients and 1 MS patient receiving RTX annually during the last dosing interval. The median number of total RTX cycles was 6 (IQR 4–7.8) in MS and 7 (IQR 5–8) in NMOSD. The duration of treatment was 30 months (IQR 20–46) in MS and 31 months (IQR 23–41) in NMOSD.

### Efficacy outcomes

#### Clinical relapses prevention

**Figure 1:**
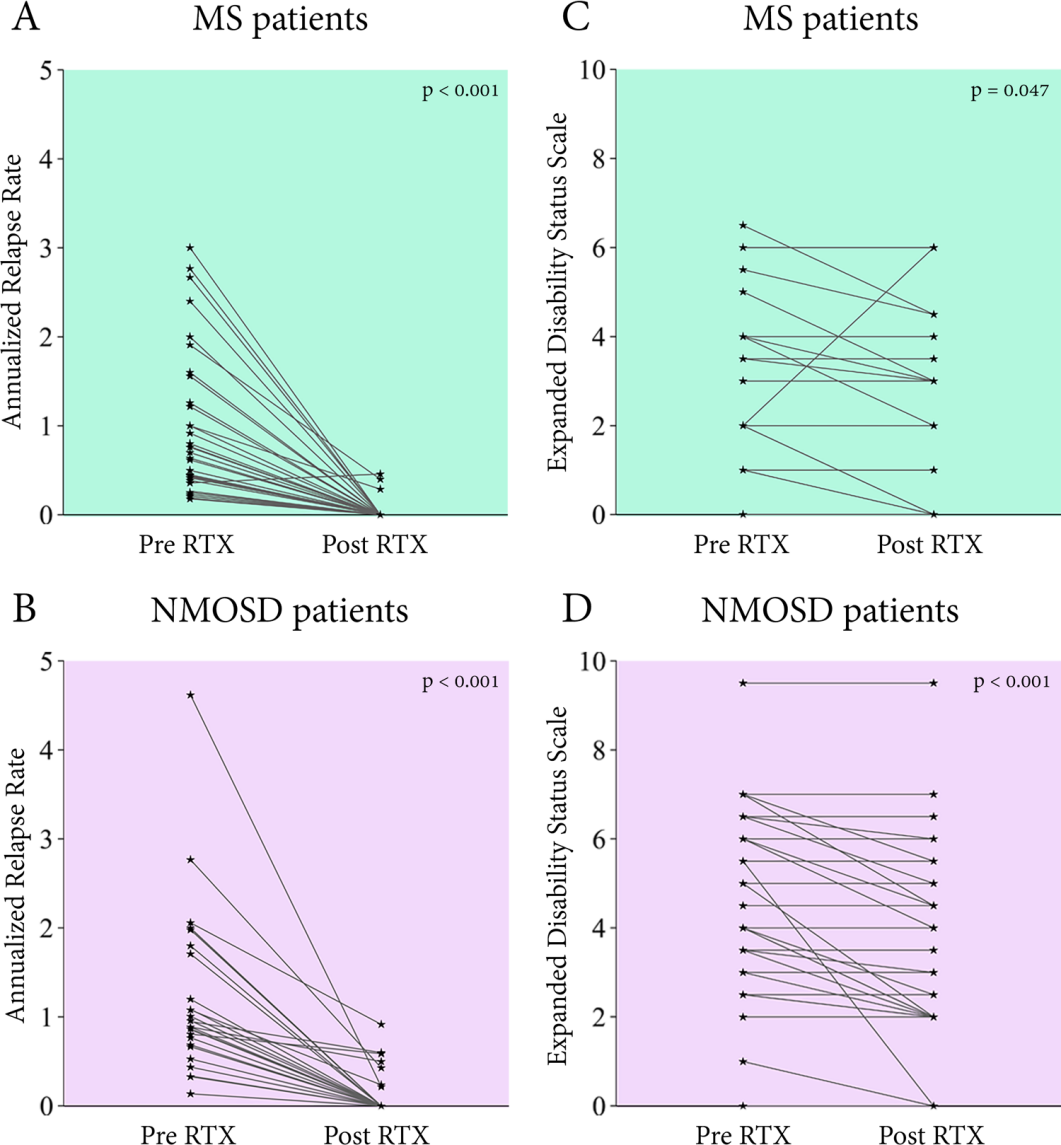
Dot plot illustrating pre and post RTX ARR among (**A**) MS patients, (**B**) NMOSD patients, and pre and post EDSS scores among (**C**) MS patients, and (**D**) NMOSD patients.

**Figure 2:**
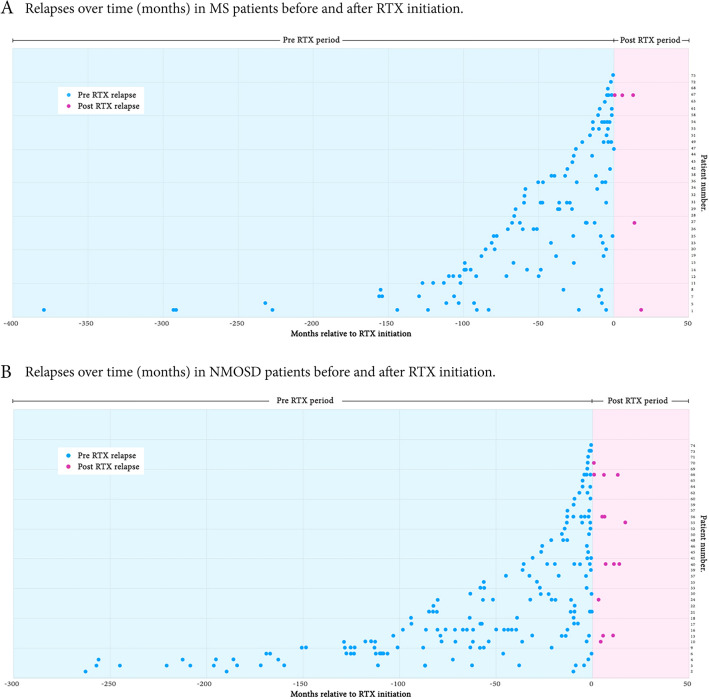
Relapses over time (months) in (**A**) MS patients and (**B**) NMOSD patients before and after RTX initiation.

After RTX initiation, the ARR and the number of relapses were significantly reduced in MS (Table 2, Fig. 1A) and NMOSD patients (Table 3, Fig. 1B). The median ARR decreased markedly from 0.77 (IQR 0.42–1.83) to 0 (IQR 0–0) (*p *< 0.001) among MS patients and from 0.92 (IQR 0.68–1.78) to 0 (IQR 0–0.17) (*p *< 0.001) among patients with NMOSD (Tables 2, 3). Thirty-three (91.7%) of MS and 31 (79.5%) of NMOSD patients had no further relapse after starting RTX, with a median follow-up period of 30 months (IQR 20–46) and 31 months (IQR 23–41), respectively (Fig. 2). Among 11 patients with post RTX relapses, 3 were MS patients and 8 were patients with NMOSD. The mean (± SD) duration from the initiation of RTX to the first subsequent relapse was 8.3 ± 3.0 months in MS and 6.8 ± 1.7 months in NMOSD. Four patients (3 with NMOSD and 1 with MS) had no further relapse after 6 months of RTX initiation. Two of those had available CD19+ B lymphocyte level data during relapse (20.9% and 5.6%). Two patients had delayed RTX doses with evidence of peripheral B lymphocyte reconstitution. The CD19+ B lymphocyte levels were 3.89% and 15.39% at the time of relapse. The other five patients had additional relapses after the initial six months after starting RTX and without delayed RTX doses. However, data on CD19+ B lymphocyte levels at the relapse time were unavailable.

**Table 2:** Efficacy of rituximab (RTX) treatment in each subgroup of Thai MS patients (n = 36).

Outcomes	Pre RTX	Post RTX	*p* value*
Multiple sclerosis (n = 36)			
ARR, times/year; median (IQR)	0.77 (0.42–1.83)	0 (0–0)	< 0.001
EDSS scores; median (IQR)	2.0 (0.0–4.0)	1.0 (0.0–3.4)	0.047
Classified by maintenance treatment regimens			
A fixed 6-month time point regimen subgroup (n = 7)			
ARR, times/year; median (IQR)	0.45 (0.42–0.70)	0 (0–0)	0.018
EDSS scores; median (IQR)	4.0 (0.0–6.0)	3.0 (0.0–6.0)	0.317
A CD19-based reinfusion regimen subgroup (n = 29)			
ARR, times/year; median (IQR)	0.92 (0.41–2.20)	0 (0–0)	< 0.001
EDSS scores; median (IQR)	2.0 (0.0–3.5)	1.0 (0.0–3.0)	0.070
Classified by history of previous DMTs or IS			
Treatment-naive subgroup (n = 11)			
Number of relapses; median (IQR)	2 (1–3)	0 (0–0)	0.003
ARR, times/year; median (IQR)	-	0 (0–0)	-
EDSS scores; median (IQR)	2.0 (0.0–4.0)	0.0 (0.0–3.0)	0.038
Treatment-experienced subgroup (n = 25)			
Number of relapses; median (IQR)	4 (2–6)	0 (0–0)	< 0.001
ARR, times/year; median (IQR)	0.64 (0.39–1.11)	0 (0–0)	< 0.001
EDSS scores; median (IQR)	2.0 (0–4.0)	1.0 (0.0–4.0)	0.340

*ARR* Annualized relapse rate, *EDSS* Expanded Disability Status Scale, *MS* Multiple sclerosis, *RTX* Rituximab.

*p value was calculated using the Wilcoxon signed-rank test.

**Table 3:** Efficacy of rituximab (RTX) treatment in each subgroup of Thai NMOSD patients (n = 39).

Outcomes	Pre RTX	Post RTX	*p* value*
NMOSD (n = 39)			
ARR^†^, times/year; median (IQR)	0.92 (0.68–1.78)	0 (0–0.17)	< 0.001
EDSS scores; median (IQR)	4.5 (3.0–6.0)	4.0 (2.0–5.5)	< 0.001
Classified by maintenance treatment regimens			
A fixed 6-month time point regimen subgroup (n = 15)			
ARR^†^, times/year; median (IQR)	0.95 (0.67–2.02)	0 (0–0.11)	0.005
EDSS scores; median (IQR)	5.0 (3.0–6.5)	4.5 (2.5–5.5)	0.042
A CD19-based reinfusion regimen subgroup (n = 24)			
ARR^†^, times/year; median (IQR)	0.92 (0.71–1.33)	0 (0–0.23)	< 0.001
EDSS scores; median (IQR)	4.3 (3.0–6.0)	3.3 (2.0–5.0)	0.002
Classified by history of previous DMTs or IS			
Treatment-naive subgroup (n = 13)			
Number of relapses; median (IQR)	2 (1–2.5)	0 (0–0.5)	0.003
ARR^†^, times/year; median (IQR)	–	0 (0–0.22)	–
EDSS scores; median (IQR)	5.0 (3.8–6.0)	4.0 (2.0–5.8)	0.027
Treatment-experienced subgroup (n = 26)			
Number of relapses; median (IQR)	4 (2–7)	0 (0–0.3)	< 0.001
ARR^†^, times/year; median (IQR)	0.89 (0.69–1.20)	0 (0–0.22)	< 0.001
EDSS scores; median (IQR)	4.3 (2.9–6.1)	3.8 (2.0–5.5)	0.003

*AQP4* aquaporin 4, *ARR* annualized relapse rate, *EDSS* Expanded Disability Status Scale, *NMOSD* neuromyelitis optica spectrum disorder, *RTX* rituximab.

*p value was calculated using the Wilcoxon signed-rank test.

^†^ARR was calculated only for NMOSD patients with a disease duration before RTX of more than 1 year. This comprised 28 NMOSD patients, with 10 in the fixed 6-month time point regimen subgroup, 18 in the CD19-based reinfusion regimen subgroup, 5 in the treatment-naive subgroup, and 23 in the treatment-experienced subgroup.

**Figure 3:**
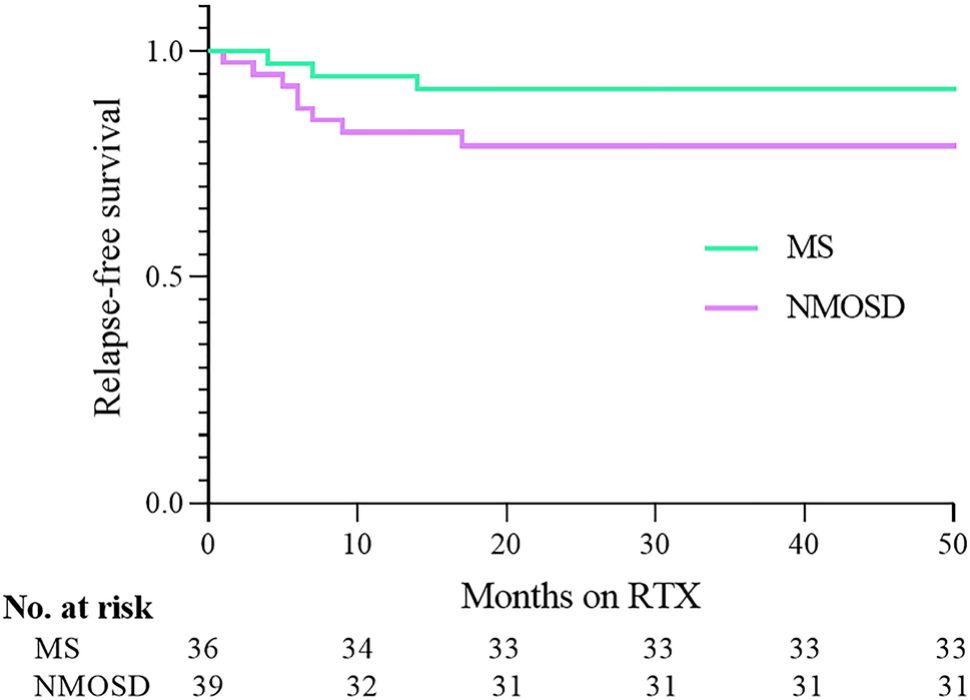
Kaplan–Meier survival analysis showing the proportion of relapse-free patients after RTX initiation in MS and NMOSD patients.

Relapse-free survival after the initiation of RTX in patients with MS and NMOSD was demonstrated by Kaplan–Meier curve (Fig. 3).

#### EDSS scores change in overall patients and stabilizing disability progression in patients with MS

EDSS scores of the majority of MS and NMOSD patients either decreased or stabilized after RTX initiation (Tables 2, 3, Fig. 1C,D). The reduction in EDSS scores reached statistical significance in both MS and NMOSD patients, decreasing from 2.0 (IQR 0.0–4.0) to 1.0 (IQR 0.0–3.4, *p *= 0.047) at the last follow-up in MS and from 4.5 (IQR 3.0–6.0) to 4.0 (IQR 2.0–5.5, *p *< 0.001) at the last follow-up in NMOSD.

Focusing on the effect on stabilizing disability progression in MS patients, 25 (69.4%) had stable EDSS scores, while 10 (27.8%) showed improved EDSS scores. In NMOSD, EDSS scores remained stable in 22 (56.4%) patients, with 17 (43.6%) showing improvement.

#### Decreasing MRI activity

Post RTX MRI data were available in 27 MS patients. Seven patients (25.9%) had new T2-weighted hyperintense lesions. Three (11.1%) patients developed new gadolinium enhancing lesions and two (7.4%) patients had enlarged T2-weighted hyperintense lesions when comparing the re-baseline MRI with the subsequent MRI.

Among the 27 MS patients with available data for the evaluation of NEDA-3, 17 patients (63%) achieved NEDA-3.

#### Subgroup analysis of efficacy outcomes

##### Maintenance treatment regimens: a fixed 6-month time point and a CD19-based reinfusion regimen

Among MS and NMOSD patients, both maintenance treatment regimens subgroups exhibited a significant decrease in ARR and the number of relapses. For MS, the median differences between pre and post RTX ARR were 0.45 (IQR 0.42–0.66) in the fixed 6-month time point subgroup and 0.92 (IQR 0.43–2.00) in the CD19-based reinfusion regimen subgroup. In NMOSD, the median differences between pre and post RTX ARR were 0.95 (IQR 0.67–1.85) in the fixed 6-month time point subgroup and 0.85 (IQR 0.35–1.33) in the CD19-based reinfusion regimen subgroup. The analysis revealed no significant differences between the two regimens in reducing ARR (*p *= 0.308 among MS and *p *= 0.302 among NMOSD). However, EDSS scores was only significantly reduced in both regimens for NMOSD patients (*p *= 0.042 in the fixed 6-month time point subgroup and *p *= 0.002 for the CD19-based reinfusion regimen subgroup) (Table 3).

##### The treatment-naïve or a treatment-experienced subgroup

The pre RTX ARR could not be rationally calculated in the treatment-naïve subgroup due to the typically short duration from onset to RTX initiation, which was often less than a year. This short duration could lead to an overestimation of the pre RTX ARR. Therefore, in the treatment-naïve subgroup, we reported the number of relapses instead. The number of relapses significantly decreased in both MS and NMOSD subgroups. The reduction in the number of relapses was more pronounced in the treatment-experienced subgroup (from 4 [IQR 2–6] to 0 [IQR 0–0], *p *< 0.001) compared to the treatment-naïve subgroup (from 2 [IQR 1–3] to 0 [IQR 0–0], *p *= 0.003) for MS. In NMOSD, the reduction was observed from 4 (IQR 2–7) to 0 (0–0.3) with a *p* value < 0.001 in the treatment-experienced subgroup and from 2 (IQR 1–2.5) to 0 (IQR 0–0.5) with a *p* value of 0.003 in the treatment-naïve subgroup. EDSS scores in MS decreased only in the treatment-naïve subgroup (*p *= 0.038). In NMOSD patients, EDSS scores decreased in both subgroups (*p *= 0.003 in the treatment-experienced subgroup and *p *= 0.027 for the treatment-naïve subgroup).

##### Extended RTX dosing interval during the maintenance phase

Post hoc subgroup analyzes were also performed and shown in Supplementary materials. 58 patients (32 MS and 26 NMOSD) received an average dosing interval of RTX of more than every 6 months during maintenance phase. The median ARR of patients who received an average RTX dosing interval for more than every 6 months was significantly reduced, decreasing from 0.86 (IQR 0.43–1.98) to 0 (IQR 0–0) in MS, and from 0.96 (IQR 0.77–1.71) to 0 (IQR 0–0.22) in NMOSD, both with *p *< 0.001. Moreover, the EDSS scores among those who received an average dosing interval of more than every 6 months decreased significantly, from 2.0 (IQR 0.0–3.9) to 1.0 (IQR 0.0–3.0) with *p *= 0.047 in MS and from 4.0 (IQR 3.0–5.6) to 3.0 (IQR 2.0–4.6) with *p *= 0.002 in NMOSD, comparing the baseline and the last follow-up EDSS scores. Therefore, extending the average dosing interval of RTX for more than every six months did not show a decrease in the efficacy of RTX.

**Figure 4:**
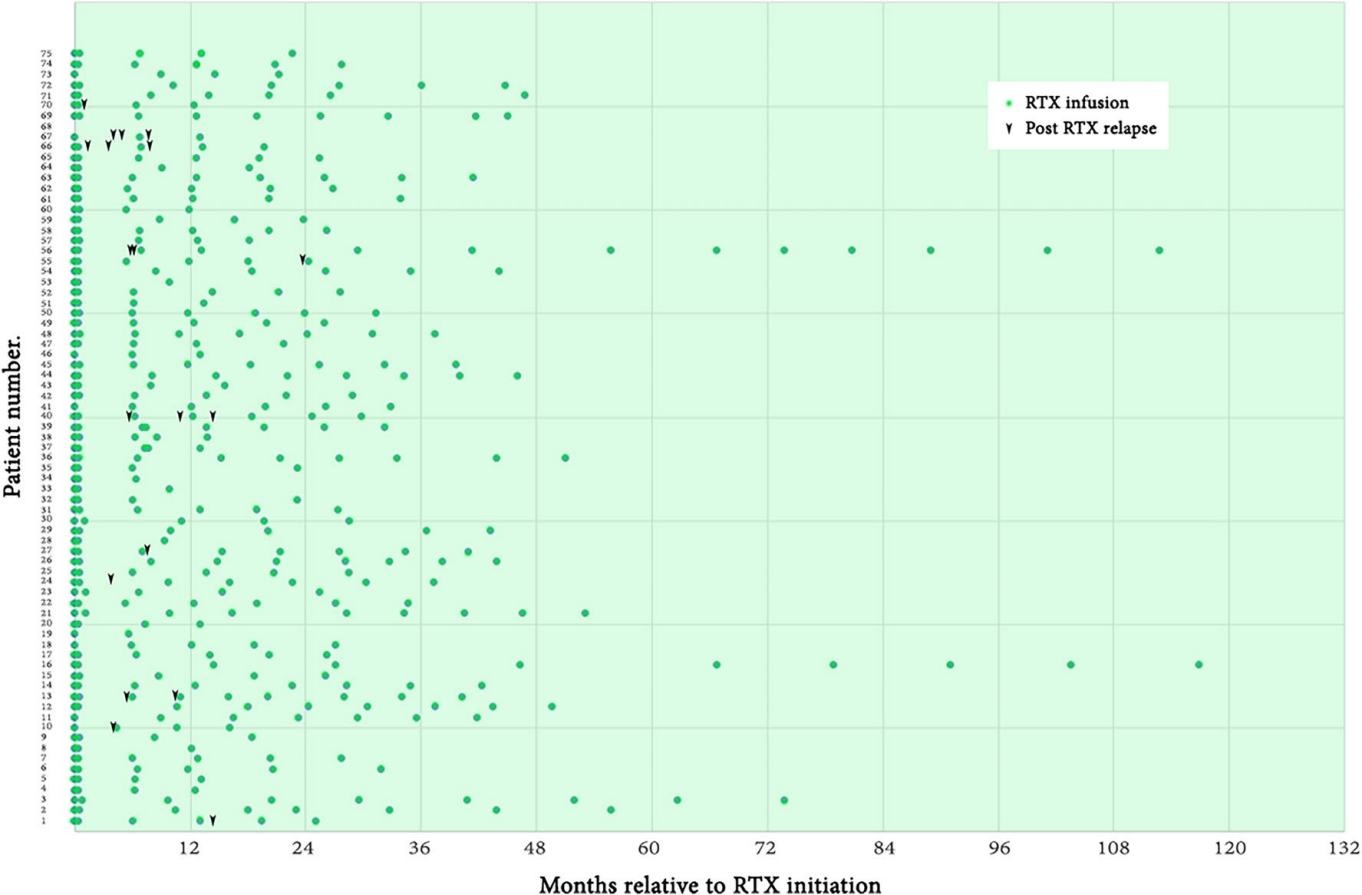
Each cycle of RTX infusion over time (months) in the overall patients.

We further stratified the average RTX dosing intervals into every 68 months, 810 months, 1012 months, and 12 months. Subgroup analyzes showed that the median ARR tended to decrease after RTX treatment among both MS and NMOSD. Moreover, there was no further relapse among six patients (1 MS and 5 NMOSD) who received RTX annually at the last dosing interval. Dosing intervals during the maintenance phase of most patients extended in subsequent cycles of RTX infusion (Fig. 4).

### Safety outcomes

Thirty-three of the 75 patients (44.0%) experienced adverse drug reactions after RTX initiation (Table 4). Infusion reactions were the most prevalent (29.3%). Among those who encountered infusion reactions, two patients had hypotension, which resolved after reducing the infusion rate. Seven patients (9.3%) had infections; urinary tract infection (n = 3), pneumonia (n = 1), upper respiratory tract infection (n = 1), acute gastroenteritis (n = 1), disseminated herpes zoster infection (n = 1). There were four patients with leukopenia (white blood cell count < 3000/μL) and five patients with grade 1 or 2 lymphopenia (absolute lymphocyte count < 1000/μL). The other two developed myalgias and arthralgia. All patients remained on RTX at the end of the study period in April 2023.

**Table 4:** Safety profile of rituximab (RTX) in Thai MS and NMOSD patients.

Adverse drug reactions	Total patients (n = 75)
Experienced adverse drug reactions, n (%)	33 (44.0)
Infusion reactions*	22 (29.3)
Infection^†^	7 (9.3)
Leukopenia, white blood cell count < 3000/μL	4 (5.3)
Lymphopenia, absolute lymphocyte count < 1000/μL	5 (6.7)
Others^‡^	2 (2.7)

*RTX* Rituximab.

*Infusion reactions included fever (n = 5), rash (n = 4), chill (n = 4), hypotension (n = 2), headache (n = 2), hypertension (n = 1), chest tightness (n = 1), palpitation (n = 1), rhinorrhea (n = 1), nausea (n = 1), hand numbness (n = 1), burning sensation both feet (n = 1).

^†^Infection included upper respiratory tract infection (n = 1), pneumonia (n = 1), urinary tract infection (n = 3), zoster infection (n = 1), acute gastroenteritis (n = 1).

^‡^Others were myalgia (*n* = 1) and arthralgia (*n* = 1).

## Discussion

While there are existing studies confirming the efficacy of RTX among individuals with MS and NMOSD, investigations into the effectiveness of RTX, particularly within South-East Asian populations, remain scarce. This is especially true concerning extended interval dosing of RTX, which provides even greater socioeconomic benefits. This retrospective multicenter observational study aimed to address this gap, showcasing the efficacy of RTX in preventing relapses by reducing ARR, decreasing the number of relapses, and increasing the proportion of relapse-free patients in both the Thai MS and NMOSD populations. RTX increased the proportion of patients who achieved NEDA-3 and was associated with stabilizing disability progression evaluated by changes in EDSS scores in MS patients. During an average median of 2.5 years of RTX therapy, 91.7% of MS and 79.5% of NMOSD patients were free of clinical attack. Furthermore, a specific regimen (two 1000 mg infusions 15 days apart as an induction regimen and one 1000 mg infusion in subsequent doses using a CD19-based reinfusion regimen or a fixed 6-month time point regimen) was prescribed in most patients. We also reported the relapse-free prescribing of the extended dosing intervals based on the CD19+ B lymphocyte monitoring of RTX. Almost half of the patients experienced adverse drug reactions, with infusion reactions as the most prevalent.

Since a pilot study on the efficacy of RTX in NMOSD patients by Cree et al. in 2005, RTX has been used off-label as a maintenance therapy in NMOSD patients^8^. A randomized, double-blind, placebo-controlled trial, RIN-1 study, in 38 NMOSD with AQP4-IgG participants showed that none of the RTX group vs 37% of the placebo group had NMOSD relapses during the 72-week study period^5^. Meta-analyzes suggested that RTX has a favorable effect on patients with NMOSD^36,37^. Furthermore, a recent retrospective study on the long-term effectiveness of RTX in NMOSD, including 111 NMOSD patients, summarized that the median ARR decreased from 1.1 before treatment to 0 after treatment, with 72% of the patients achieving relapse-free during the median follow-up of 3.7 years^9^. The reduction in the mean EDSS scores was statistically significant in all meta-analyses^6,36,37^. This study highlighted the efficacy of RTX in preventing NMOSD relapse and reducing EDSS scores, with a high proportion (79.5%) of Thai NMOSD patients achieving relapse-free after the initiation of RTX.

As in MS, apart from the cell-mediated immune response, B lymphocytes are critical to the pathogenesis of MS^10^. A randomized controlled trial demonstrated a decrease in inflammatory brain lesions and clinical relapse for 48 weeks in 69 patients receiving a single course of RTX compared to 35 patients receiving a placebo^38^. Moreover, a large multicenter cohort (n = 822) in Sweden reported a low ARR ratio of 0.044 and a constant median EDSS scores over a follow-up period of almost two years in the RRMS population treated with a dose of 500–1000 mg of RTX every 6–12 months^11^. The previous meta-analysis also demonstrated the efficacy of RTX in improving relapse and disability conditions in patients with MS, with the overall reduction in ARR and EDSS scores of 1.00 (95% confidence interval [CI] 0.83–1.17) and 0.62 (95% CI 0.20–1.04), respectively^39^. According to the study findings, the efficacy of RTX among Thai MS patients was the same as in previous studies. NEDA-3 was achieved in 63% of MS patients.

Focusing on baseline disease activity of the study patients, there was evidence of disease activity 1 year prior to RTX initiation; the median number of relapses was 1 (IQR 0.3–1) in MS and 1 (IQR 1–2) in NMOSD, and a 41.9% proportion of MS patients with gadolinium enhancing lesions. Moreover, data from treatment-naïve and treatment-experienced subgroup analysis found the efficacy of RTX in preventing relapses in both subgroups. Therefore, RTX was effective even in those with recent evidence of disease activity and those with DMTs/IS failure.

Several retrospective studies in MS and NMOSD patients demonstrated an indistinguishable efficacy of extended RTX dosing intervals under CD19+ or CD27+ B-cell level monitoring^7,25,27,28,40^. A meta-analysis evaluating the efficacy and safety of different doses of RTX in NMOSD showed that an initial dose of 100 mg RTX intravenous infusion per week for three consecutive weeks resulted in the same efficacy as other conventional dose regimens^41^. In the current study, the majority of the patients received a CD19-based reinfusion regimen with a median average dosing interval of 6.8 months (IQR 6.3–7.5) in MS patients and 6.4 months (IQR 6.0–7.8) in NMOSD patients. The subgroup analyses demonstrated that the efficacy of RTX in preventing relapses was robust in both a fixed 6-month time point and a CD19-based reinfusion regimen subgroup. Extending the dosing interval of RTX for more than every six months did not diminish the efficacy of RTX, even in some patients with a dosing interval of 12 months. Extending the dosing interval of RTX is beneficial in 2 aspects. Risks associated with continued immunosuppression, including hypogammaglobulinemia, infections, or malignancy, could be reduced. In addition, reducing dosing frequency gives socioeconomic benefits, especially in low- to middle-income countries.

Among eleven patients with post RTX relapse (3 MS patients and 8 NMOSD), four patients (1 MS and 3 NMOSD) had evidence of peripheral B-lymphocyte reconstitution during the relapse period. Data on CD19+ B lymphocyte levels at relapse period were unavailable in the remaining seven patients, limiting the comprehensive evaluation of peripheral B lymphocyte reconstitution in this study. However, it should be noted that the mean (± SD) duration from the initiation of RTX to the first subsequent relapse was 8.3 ± 3.0 months in MS and 6.8 ± 1.7 months in NMOSD. Remarkably, 36.4% of the patients who experienced post-RTX relapse did not have any further relapses after the initial six months. These findings suggest the potential benefit of gradually tapering off oral corticosteroids over at least a 2-month period while RTX achieves its immunosuppressive effects.

For the safety outcomes of RTX among Thai MS and NMOSD patients, almost half of the patients had adverse drug reactions after RTX initiation. Infusion reactions and infection were the most frequent. Among those experiencing infection, urinary tract infection was the leading cause. A study reported that 19.3% of patients with normal IgG levels within the reference range prior to RTX treatment developed mild to severe hypogammaglobulinemia after receiving RTX^42^. To mitigate the long-term adverse drug reactions of anti-CD20 therapy, such as hypogammaglobulinemia and infection, the implementation of extended dosing intervals has been considered. However, further investigation is needed to explore this issue in depth.

This study has certain limitations. First, the study was a retrospective review of the chart, so there may be some data on adverse drug reactions that were not documented in the medical record. Second, CD19+ B lymphocyte levels were not tested at regular intervals. Also, some patients did not have CD19+ B lymphocyte level testing before each cycle of RTX or during relapse. Thus, peripheral B-lymphocyte reconstitution could not be completely evaluated. Third, the study had a limited sample size. However, we strongly believe that this study holds significant value for low- to middle-income countries. The findings of this study indicate that RTX could serve as a cost-effective therapy, either as a first-line agent or as a rescue therapy option, for MS and NMOSD patients in these resource-constrained settings. For further studies, a randomized controlled trial using the extended dosing interval of RTX in MS and NMOSD patients should be conducted.

## Conclusion

This study shows that RTX is an effective treatment for MS and NMOSD patients. Relapses were markedly lowered after RTX initiation. No serious adverse drug reactions were observed. This study highlights the efficacy of extended RTX dosing intervals, which is advantageous for cost-effectiveness. We encourage the addition of RTX as a therapeutic option for MS and NMOSD patients in low- to middle-income countries, as it would be beneficial.

### Supplementary Information

Supplementary Tables.

## Data Availability

Data supporting the findings are available upon request from the corresponding authors.
