# Supplementary material for: Efficacy and safety of rituximab in multiple sclerosis and neuromyelitis optica spectrum disorder
Source: Sci Rep. 2024 Feb 12;14:3503. doi: 10.1038/s41598-024-53838-y (PMC10861443; doi:10.1038/s41598-024-53838-y)
Supplement: Supplementary file 1 — Supplementary Tables. [file 41598_2024_53838_MOESM1_ESM.docx]

**Supplementary Table 1** Efficacy of rituximab treatment in each subgroup classified by average dosing interval of Thai MS patients.

| **Outcomes** | **Pre RTX** | **Post RTX** | **p-value*** |
| --- | --- | --- | --- |
| **Patients receiving rituximab every 6 months** (n=4) | | | |
| ARR, times/year; median (IQR) | 0.42 (0.31-0.63) | 0 (0-0) | 0.066 |
| EDSS scores; median (IQR) | 3.0 (0.0-6.0) | 3.0 (0.0-6.0) | 1.000 |
| **Patients receiving an extended dosing interval of rituximab for more than 6 months** (n=32) | | | |
| ARR, times/year; median (IQR) | 0.86 (0.43-1.98) | 0 (0-0) | < 0.001 |
| EDSS scores; median (IQR) | 2.0 (0.0-3.9) | 1.0 (0.0-3.0) | 0.047 |
| **Patients receiving rituximab every 6-8 months** (n=26) | | | |
| ARR, times/year; median (IQR) | 0.96 (0.44-1.93) | 0 (0-0) | < 0.001 |
| EDSS scores; median (IQR) | 2.0 (0.0-4.0) | 2.0 (0.0-3.6) | 0.071 |
| **Patients receiving rituximab every 8-10 months** (n=5) | | | |
| ARR, times/year; median (IQR) | 0.64 (0.35-7.39) | 0 (0-0) | 0.043 |
| EDSS scores; median (IQR) | 0.0 (0.0-1.0) | 0.0 (0.0-1.0) | 1.000 |
| **Patients receiving rituximab every 10-12 months** (n=1) | | | |
| ARR, times/year; median (IQR) | 0.25 | 0 | - |
| EDSS scores; median (IQR) | 2.0 | 0.0 | - |

^*^p-value was calculated using the Wilcoxon signed-rank test
Abbreviations: AQP4, aquaporin 4; ARR, annualized relapse rate; EDSS, Expanded Disability Status Scale; NMOSD, neuromyelitis optica spectrum disorder; RTX, rituximab.

**Supplementary Table 2** Efficacy of rituximab treatment in each subgroup classified by average dosing interval of Thai NMOSD patients.

| **Outcomes** | **Pre RTX** | **Post RTX** | **p-value*** |
| --- | --- | --- | --- |
| **Patients receiving rituximab every 6 months** (n=13) | | | |
| ARR^†^, times/year; median (IQR) | 0.89 (0.67-1.90) | 0 (0-0.30) | 0.008 |
| EDSS scores; median (IQR) | 5.5 (3.8-6.8) | 5.5 (2.8-6.3) | 0.042 |
| **Patients receiving an extended dosing interval of rituximab for more than 6 months** (n=26) | | | |
| ARR^†^, times/year; median (IQR) | 0.96 (0.77-1.71) | 0 (0-0.22) | < 0.001 |
| EDSS scores; median (IQR) | 4.0 (3.0-5.6) | 3.0 (2.0-4.6) | 0.002 |
| **Patients receiving rituximab every 6-8 months** (n=16) | | | |
| ARR^†^, times/year; median (IQR) | 1.08 (0.81-1.71) | 0 (0-0.24) | 0.003 |
| EDSS scores; median (IQR) | 3.5 (3.0-5.9) | 3.0 (2.0-4.0) | 0.012 |
| **Patients receiving rituximab every 8-10 months** (n=4) | | | |
| ARR^†^, times/year; median (IQR) | 0.86 (0.77-0.89) | 0 (0-0.5) | 0.109 |
| EDSS scores; median (IQR) | 4.0 (2.9-5.1) | 3.3 (2.1-5.1) | 0.180 |
| **Patients receiving rituximab every 10-12 months** (n=5) | | | |
| ARR^†^, times/year; median (IQR) | 0.61 (0.19-3.68) | 0 (0-0.17) | 0.068 |
| EDSS scores; median (IQR) | 4.5 (2.0-5.8) | 4.5 (1.0-5.0) | 0.180 |
| **Patients receiving rituximab every 12 months** (n=1) | | | |
| ARR^†^, times/year | 1.98 | 0 | - |
| EDSS score | 7.0 | 7.0 | - |

^*^p-value was calculated using the Wilcoxon signed-rank test
^†^ARR was calculated only for NMOSD patients with a disease duration before RTX of more than 1 year. This group comprised 9 patients with an average dosing interval of every 6 months, 19 patients with an average dosing interval of more than 6 months (extended dosing interval), 11 patients with an average dosing interval of every 6-8 months, 3 patients with an average dosing interval of every 8-10 months, 4 patients with an average dosing interval of every 10-12 months, and 1 patient with an average dosing interval of every 12 months
Abbreviations: AQP4, aquaporin 4; ARR, annualized relapse rate; EDSS, Expanded Disability Status Scale; NMOSD, neuromyelitis optica spectrum disorder; RTX, rituximab.
